# Novel Therapeutic Insights Into Alzheimer's Disease: Glymphatic System and Meningeal Lymphatic Vessels

**DOI:** 10.1111/acel.70699

**Published:** 2026-09-10

**Authors:** Bingbing Song, Wei Wang, Siqi Liu, Xi Jin, Yan Qi, Mingquan Li, Dongjie Yue, Yu Liu, Xiangqi Li, Lei Yin, Lina Feng

**Affiliations:** ^1^ Department of Neurology The Third Affiliated Hospital of the Changchun University of Chinese Medicine Changchun Jilin Province People's Republic of China; ^2^ Department of Intensive Care Unit The Affiliated Hospital to Changchun University of Chinese Medicine Changchun Jilin Province People's Republic of China; ^3^ Department of Operating Room The Second Affiliated Hospital of Shandong First Medical University, Shandong First Medical University & Shandong Academy of Medical Sciences Taian Shandong Province People's Republic of China; ^4^ Department of General Surgery The 32183 Military Hospital of PLA Baicheng Jilin Province People's Republic of China; ^5^ Department of Traditional Chinese Medicine The Second Affiliated Hospital of Shandong First Medical University, Shandong First Medical University & Shandong Academy of Medical Sciences Taian Shandong Province People's Republic of China; ^6^ Department of Anesthesiology, Shanghai Pulmonary Hospital, School of Medicine Tongji University Shanghai People's Republic of China

**Keywords:** Alzheimer's disease, Aβ, blood–brain barrier, cerebrospinal fluid, glymphatic system, interstitial fluid, meningeal lymphatic vessels

## Abstract

With the acceleration of global aging, the relationship between Alzheimer's disease (AD) and the glymphatic system (GS) has become a research hotspot in the field of neuroscience in recent years. Traditionally, the central nervous system was thought to lack a lymphatic system; however, research over the past decade has overturned this view. Studies have revealed the existence of GS and meningeal lymphatic vessels (mLVs) in the brain, which clear metabolic waste (such as amyloid‐β and tau proteins) through the exchange of cerebrospinal fluid (CSF) and interstitial fluid (ISF). Dysfunction of the GS can lead to abnormal deposition of pathological proteins, which may trigger or exacerbate AD. Currently, the association between the GS and AD treatment mainly focuses on drug development (such as small molecules that promote glymphatic circulation), physical therapies (such as 40 Hz photoacoustic therapy), and surgical interventions (primarily deep cervical lymphatic‐vein anastomosis and cranial bone maneuver), all of which are under exploration. With the precision of diagnostic technologies and the targeting of therapeutic methods, future intervention strategies surrounding GS may become a significant breakthrough in overcoming the treatment impasse of AD, bringing new hope to tens of millions of AD patients worldwide.

## Graphical abstract

The identification of the glymphatic system (GS) and meningeal lymphatic vessels (mLVs) challenges the century‐old “immune‐privileged” dogma of the central nervous system (CNS), establishing a critical mechanistic link between impaired waste clearance (Aβ/tau) and the pathogenesis of Alzheimer's disease (AD). Targeted modulation of the glymphatic pathway represents a transformative precision medicine strategy with immense clinical potential to reverse the pathological cascade of AD and break the current therapeutic impasse.

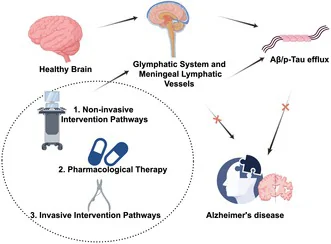

**Abbreviations**
AD: Alzheimer's disease
APOE: apolipoprotein E
APP: amyloid precursor protein
APP/PS1: APPswe/PSEN1dE9
AQP4: aquaporin‐4
Aβ: amyloid‐β
BBB: blood–brain barrier
CAA: cerebral amyloid angiopathy
CDR‐SB: Clinical Dementia Rating Scale‐Sum of Boxes
CNS: central nervous system
CSF: cerebrospinal fluid
CSVD: cerebral small vessel disease
CTBS: continuous theta‐burst stimulation
dcLNs: deep cervical lymph nodes
dcLVA: deep cervical lymphatic‐vein anastomosis
DMT: disease‐modifying therapies
DTI‐ALPS: diffusion tensor image analysis along the perivascular space
EEG: electroencephalogram
FDA: Food and Drug Administration
FUS‐MB: focused ultrasound treatment in combination with microbubbles
GS: glymphatic system
HUCBCs: human umbilical cord blood cells
IL: interleukin
iNPH: idiopathic normal pressure hydrocephalus
iRBD: isolated REM sleep behavior disorder
ISF: interstitial fluid
L‐NBP: L‐3‐n‐butylphthalide
mLVs: meningeal lymphatic vessels
MRI: magnetic resonance imaging
OSA: obstructive sleep apnea
PND: perioperative neurocognitive disorder
PUFAs: polyunsaturated fatty acids
PVS: perivascular space
REM: rapid eye movement
rTMS: repetitive transcranial magnetic stimulation
SD: sleep deprivation
TVS: transcranial vibration stimulation
VEGF‐C: vascular endothelial growth factor‐C
VIP: Vasoactive Intestinal Peptide
WMH: white matter hyperintensities

## 1 Introduction

Alzheimer's disease (AD), as a typical representative of degenerative diseases of the central nervous system (CNS), exhibits a significant correlation with the process of population aging. Globally, the prevalence among individuals aged 65 and older has reached 5%–8%, and as the proportion of the elderly population continues to rise, AD is gradually evolving into a major challenge in the field of global public health in the 21st century (GBD 2019 Dementia Forecasting Collaborators 2022). AD is the most common type of dementia, and its core pathological features are primarily characterized by the extracellular accumulation of amyloid‐β (Aβ) plaques and the intracellular hyperphosphorylated tau protein that forms neurofibrillary tangles (Sighencea et al. 2024). This irreversible neurodegenerative process leads to progressive cognitive impairment and behavioral abnormalities in patients, clinically manifesting as multidimensional deficits in memory, language, and executive function (Zhang, Zhu, et al. 2025). As the disease progresses, patients gradually lose basic activities of daily living, resulting in a significantly shortened lifespan and an increased demand for long‐term care and medical expenditures, which impose a heavy burden on families and the socioeconomic system (Alzheimer's Association 2024). The glymphatic system (GS) is an important pathway for the clearance of metabolic waste products in the brain, and its discovery provides new strategies for the prevention and treatment of AD. The lymphatic system is a unidirectional channel with a blind end that maintains homeostasis by clearing excess interstitial fluid (ISF), macromolecules, and immune cells from the interstitial space into the bloodstream (Alitalo 2011). Previously, it was believed that the CNS was the only organ system lacking lymphatic vessels to assist in the clearance of interstitial metabolic waste products. However, this perspective does not adequately explain how the large amounts of metabolic products generated by brain cells, which consume 20%–25% of the total energy of the human body, are cleared and how fluid homeostasis is maintained. With the advancement of research on the clearance of metabolic substances in the brain, it has been discovered that the CNS has a GS, which plays an important role in the occurrence and development of neurodegenerative diseases, age‐related changes in the brain, and traumatic brain injuries (Iliff et al. 2012). The GS consists of aquaporin‐4 (AQP4) located on the end feet of astrocytes and the perivascular space (PVS), playing a crucial role in facilitating the exchange and flow of cerebrospinal fluid (CSF) and ISF. CSF enters the brain parenchyma through the PVS adjacent to cortical arteries and penetrating arterioles, enabling material exchange between CSF and ISF, while metabolic waste is cleared through the PVS of small veins, ultimately transporting waste to the deep cervical lymph nodes (dcLNs) and peripheral lymphatic system (Szczygielski et al. 2021). The GS can eliminate soluble brain metabolites such as Aβ, tau protein, α‐synuclein, lipids, pro‐inflammatory cytokines, and neurotoxic solutes (Iliff et al. 2012; Gao et al. 2015; Kriegel et al. 2018; Lundgaard et al. 2015; Thrane et al. 2013; Murlidharan et al. 2016).

Dysfunction of the GS can lead to a decreased clearance rate of soluble brain metabolites. Boespflug et al. (Boespflug et al. 2018) measured the PVS in postmortem pathological samples from participants with intact cognition or those suffering from AD or mixed dementia (vascular lesions accompanied by AD). The results indicated that the PVS were significantly enlarged in the AD and mixed dementia groups compared to cognitively intact subjects, and this enlargement was associated with tau and Aβ pathology (Boespflug et al. 2018). Hoshi et al. (2012) discovered through immunohistochemistry that the expression of AQP4 in AD patients is associated with the distribution of Aβ_42_‐ or Aβ_40_‐positive senile plaques. Several animal studies have confirmed that the occurrence and development of AD are closely related to dysfunction of GS. Xu et al. (Xu et al. 2015) found that the deletion of AQP4 exacerbates cognitive impairments in 12‐month‐old APPswe/PSEN1dE9 (APP/PS1) mice, accompanied by the accumulation of Aβ protein in the hippocampus and cortex, cerebrovascular amyloid pathology, and the loss of synaptic proteins and brain‐derived neurotrophic factor. Using molecular imaging technology with water molecule tracking, Igarashi et al. (Igarashi et al. 2014) quantitatively analyzed the influx of water into the CSF of APP/PS1 mice, revealing that the influx of water into the CSF is significantly restricted, with a degree nearly consistent with that in AQP4 knockout mice. Iliff et al. (Iliff et al. 2012) employed in vivo two‐photon imaging technology with small fluorescent tracers and found that animals lacking the water channel AQP4 exhibited a slowdown in CSF flow through the GS and a reduction of approximately 70% in interstitial solute clearance. Additionally, the absence of the AQP4 gene inhibited the clearance of soluble Aβ protein. These findings indicate that the absence of AQP4 can lead to impaired clearance of Aβ protein and plays an important role in the formation of senile plaques. Deep cervical lymphatic‐vein anastomosis (dcLVA) is a novel therapeutic method for AD pioneered by Chinese scholars. This technique utilizes microsurgical methods to anastomose deep cervical lymphatics with adjacent veins, thereby reconstructing the lymphatic‐venous pathway to improve cerebral lymphatic circulation and treat AD. The procedure is minimally invasive, completed through two small incisions in the neck, resulting in minimal trauma and rapid recovery. Postoperative CSF circulation dynamics improve, enhancing the function of the GS, and patients experience a certain degree of alleviation in cognitive functions (Fu 2024). However, dcLVA is still in the preliminary clinical exploration stage. The applicable population and screening criteria remain to be clarified, and the small sample size limits the results, leaving the potential risks unknown. Further research is needed to explore these issues through larger sample sizes and long‐term follow‐ups.

In summary, the GS provides a new perspective for AD research, with potential implications for the pathological mechanisms and treatment of AD, bringing new hope and possibilities for prevention and treatment. In‐depth studies on the correlation between the GS and AD may lead to the development of more effective diagnostic and therapeutic methods for AD patients.

## 2 The Current Status of Emerging Disease‐Modifying Therapies for AD

Currently, the treatment drugs for AD are mainly divided into two categories: symptomatic treatment drugs and disease‐modifying therapies (DMT). The symptomatic treatment drugs for AD approved by the U.S. Food and Drug Administration (FDA) primarily include cholinesterase inhibitors (such as donepezil, rivastigmine, and galantamine) and N‐methyl‐D‐aspartate receptor antagonists (such as memantine). However, these drugs are unable to halt or reverse the progression of the disease (Kshirsagar et al. 2021; Scheltens et al. 2021), DMT intervenes in the pathophysiological processes of AD (Table 1).

**TABLE 1:** Comparison table of mechanism of action, dosing regimen, clinical efficacy and safety of Aβ/Tau‐targeted drugs.

Name	Mechanism of action	Dosing regimen	Indication/inclusion criteria	Efficacy	Adverse events	References
Lecanemab	Binds to soluble amyloid‐β (Aβ) in the brain and promotes Aβ clearance	Intravenous injection, once every 2 weeks	Patients with early‐stage Alzheimer's disease (AD).	Improves scores on relevant scales and reduces cerebral Aβ levels.	Amyloid‐Related Imaging Abnormalities (ARIA).	Mahase (2024)
Donanemab	Specifically bind to cerebral amyloid plaques and promote plaque clearance	Intravenous injection, once every 4 weeks	Early symptomatic AD, including Mild Cognitive Impairment (MCI) or mild dementia.	Positive trial outcome: 23 out of 24 prespecified endpoints achieved statistical significance.	Amyloid‐Related Imaging Abnormalities of edema or effusion (ARIA‐E).	Sims et al. (2023)
Solanezumab	Targeting soluble Aβ in the brain to promote its clearance and block Aβ‐mediated synaptic toxicity	Intravenous injection, once every 4 weeks	Mild dementia due to AD	Negative outcome, failing to meet the primary endpoint	Favorable safety profile; extremely low incidence of cerebral edema or effusion lesions.	Honig et al. (2018)
Gantenerumab	Exhibits the highest affinity for aggregated Aβ, promoting amyloid plaque clearance by binding to this target.	Intravenous injection, once every 2 weeks	Early AD (mild cognitive impairment/mild dementia)	Negative outcome; no statistically significant differences in Clinical Dementia Rating Scale‐Sum of Boxes (CDR‐SB) changes vs. placebo in both trials.	ARIA‐E	Bateman et al. (2023)
Valiltramiprosate	Anti‐Aβ oligomer agents to exert disease‐modifying effects by inhibiting Aβ oligomer formation	Administered orally; twice‐daily with the morning and evening meals	Apolipoprotein E (APOE) 4 carriers; patients with early‐stage AD	Arrested the progressive decline in cerebrospinal fluid (CSF) Aβ_42_ level and plasma Aβ_42_/Aβ_40_ ratio, and stabilized Rey Auditory Verbal Learning Test (RAVLT).	No amyloid‐related imaging abnormalities (ARIA) reported	Hey et al. (2024, 2025)
Gosuranemab	A monoclonal antibody targeting the N‐terminus of tau may prevent or reverse its abnormal phosphorylation and aggregation.	Intravenous injection, once every 4 weeks.	MCI or mild AD	All doses caused significant reductions in the CSF levels of unbound N‐terminal tau. No significant effects were identified in cognitive and functional tests	No significant adverse reactions were observed.	Shulman et al. (2023)
Semorinemab	An Anti‐tau monoclonal antibody, which may prevent the abnormal phosphorylation of tau protein.	Intravenous injection, once every 4 weeks.	Mild‐to‐moderate AD	Statistically significant improvement was observed in Alzheimer's Disease Assessment Scale‐Cognitive Subscale (ADAS‐Cog); No statistically significant therapeutic effects were observed in Alzheimer's Disease Cooperative Study‐Activities of Daily Living (ADCS‐ADL), Mini‐Mental State Examination (MMSE), or CDR‐SB.	No significant adverse reactions were observed.	Fu (2024)
Zagotenemab	By targeting and binding to misfolded, extracellular, aggregated tau protein, it may prevent its further aggregation, promote its clearance, or inhibit its propagation between neurons.	Intravenous injection, once every 4 weeks.	Early symptomatic AD	No evidence of clinical disease modification (cognitive/functional) was demonstrated.	Elevated adverse event rate (85.1%) observed in treated patients.	Kshirsagar et al. (2021)
Tilavonemab	Monoclonal antibody targeting the N‐terminus of human tau protein.	Intravenous injection, Loading doses on Days 1, 15, 29; then every 28 days.	Progressive Supranuclear Palsy (PSP)	No significant difference in Progressive Supranuclear Palsy Rating Scale (PSPRS) total score change.	The safety profile was similar across all groups.	Hoglinger et al. (2021)

In June 2021, the FDA granted accelerated approval for the DMT aducanumab for the treatment of AD, which is a recombinant monoclonal antibody that reduces Aβ. The results of its Phase 3 clinical trials indicated that it could reduce brain Aβ plaques, but the long‐term clinical benefits have not been sufficiently validated (Rabinovici 2021; Hershey and Tarawneh 2021), and its high cost is burdensome for patients (Heidebrink and Paulson 2024). Additionally, common adverse reactions during treatment include brain edema and intracerebral hemorrhage, and imaging may show amyloid‐related imaging abnormalities. In July 2023, Lecanemab became the first anti‐Aβ monoclonal antibody approved by the U.S. FDA through the traditional pathway. It promotes the clearance of soluble Aβ in the brain by binding to it. Furthermore, subgroup analyses from Phase 3 clinical trials indicated that the therapeutic effects of Lecanemab were not significant in women and patients under the age of 65. The severity of its adverse reactions outweighed the minimal benefits it provided (Mahase 2024). Donanemab is an immunoglobulin G1 monoclonal antibody that removes amyloid plaques in the brain by promoting microglial‐mediated phagocytosis (Sims et al. 2023). However, during its use, approximately 37% of patients in the treatment group experienced amyloid‐related imaging abnormalities, with a higher incidence observed particularly in the population carrying the apolipoprotein E (APOE) ε4 homozygous genotype (Manly and Deters 2023). Unfortunately, another drug, Solanezumab, failed to demonstrate improvements in cognitive function for patients with mild to moderate AD across multiple large clinical trials (Sperling et al. 2023). While there was some improvement in AD‐related biomarkers in preventive studies, the enhancement of cognitive function remained limited (Honig et al. 2018; Doggrell 2024). Gantenerumab is a fully human anti‐Aβ immunoglobulin G1 monoclonal antibody administered subcutaneously, which exhibits a high affinity for aggregated Aβ, including oligomers, protofibrils, and plaques. Results from Phase III clinical trials indicate that Gantenerumab does not show significant improvement in the primary outcome measure (Clinical Dementia Rating Scale‐Sum of Boxes score), and patients carrying the APOE ε4 allele have a higher incidence of cerebral edema (Bateman et al. 2023). Valiltramiprosate (ALZ‐801) is an orally administered, well‐tolerated synthetic valine‐conjugate prodrug of tramiprosate. Its active metabolites include tramiprosate and 3‐sulfopropanoic acid. The proposed mechanism of action involves multiligand binding to Aβ_42_, which stabilizes amyloid monomers and prevents peptide aggregation and oligomerization (Lee et al. 2024). A two‐year Phase 2 clinical trial, which primarily enrolled patients carrying the APOE ε4 allele and in the early stages of AD, demonstrated that valiltramiprosate treatment was associated with a slowing of the decline in CSF Aβ_42_ levels, stabilization of the plasma Aβ_42_/Aβ_40_ ratio, and preservation of memory function in patients (Hey et al. 2024).

Reducing tau protein phosphorylation and its tangles is another key target in drug development. Various monoclonal antibodies targeting different sites on tau protein have been developed and are undergoing Phase II clinical trials, including drugs primarily designed for the N‐terminus of tau protein such as Gosuranemab (Shulman et al. 2023), Semorinemab (Monteiro et al. 2023), Zagotenemab (Fleisher et al. 2024), and Tilavonemab. Although gosuranemab at all tested doses induced a significant reduction in the level of unbound N‐terminal tau protein in CSF, no significant cognitive improvement was observed clinically (Shulman et al. 2023). Although semorinemab showed a significant effect on the cognitive endpoint (Alzheimer's Disease Assessment Scale‐Cognitive Subscale [ADAS‐Cog] 11), such an effect did not extend to improvements in functional (Alzheimer's Disease Cooperative Study‐Activities of Daily Living [ADCS‐ADL]) or global (Mini‐Mental State Examination [MMSE], Clinical Dementia Rating Scale‐Sum of Boxes [CDR‐SB]) outcomes (Monteiro et al. 2023), there was no evidence that zagotenemab could slow clinical cognitive and functional decline, nor did it exhibit any superiority over the placebo. Neither flortaucipir Positron Emission Tomography (PET), volumetric Magnetic Resonance Imaging (MRI), nor plasma Neurofilament light chain (NfL) analyses demonstrated a therapeutic effect (Fleisher et al. 2024). With respect to tilavonemab, no significant difference was noted in the change in Progressive Supranuclear Palsy Rating Scale (PSPRS) total score from baseline to Week 52 between the tilavonemab and placebo groups (Hoglinger et al. 2021).

However, these have failed to effectively inhibit tau protein tangles or improve patients' cognitive function, ultimately resulting in all trials being declared unsuccessful. Therefore, we must continue our relentless efforts to find new breakthroughs and provide more and better treatment options for AD patients.

## 3 Overview of the Glymphatic System

### 3.1 Discovery of the Glymphatic System

Lymphatic circulation exists in most human tissues, critical for metabolic waste clearance, fluid balance maintenance, and regulation of immune cell interactions and substance transport (Davis et al. 2025). Traditionally, the brain was considered an “immune‐privileged” organ; the CNS primarily relies on CSF circulation and the blood–brain barrier (BBB) for metabolite clearance (Nikolenko et al. 2020), with long‐standing controversy over the existence of an intracerebral lymphatic system. In 2012, Iliff first confirmed the GS in mice: injecting a fluorescent tracer into the cisterna magna revealed that subarachnoid CSF enters the brain parenchyma via perivascular pathways, exchanges substances with ISF, and exits through venous pathways (Iliff et al. 2012). Notably, fluid transport was significantly impaired in AQP4‐knockout mice. Given that this CSF‐ISF clearance pathway depends on astrocyte endfoot AQP4 and functions similarly to the peripheral lymphatic system, it was named the GS. In 2013, Iliff, Lee, et al. (2013) first verified GS structure and function using contrast‐enhanced MRI at the imaging level. Although constant CNS immune surveillance within the meningeal compartment is now recognized, the mechanisms regulating immune cell entry/exit from the CNS remain unclear.

In the search for T cell entry/exit pathways in the meninges, Louveau et al. (2015) identified functional mLVs in dural sinuses in 2015. These vessels express typical lymphatic endothelial cell molecular markers, transport CSF‐derived fluid and immune cells, and connect to dcLNs, further refining the cerebral lymphatic network. In 2019, Ahn et al. (2019) confirmed mLVs as the primary pathway for clearing large molecules from CSF by visualizing their anatomical locations and morphological features, highlighting the critical role of basal mLVs in CSF drainage (distinct from dorsal mLVs, basal mLVs associate with subarachnoid lymphatics and capillaries). In 2022, Albayram et al. (2022) first visualized human meningeal lymphatic structures via 3D T2‐fluid‐attenuated inversion recovery MRI, distributed along the dorsal dural venous sinuses and ventral cranial nerves‐with direct connections to cervical lymph nodes. Age‐related changes were also identified: cervical lymph node atrophy and thickening of dorsal/ventral lymphatic channels, suggesting potential age‐related decline in cerebral lymphatic outflow function. This research fills a neuroanatomical gap and provides key technical support for GS functional assessment and neurodegenerative disease research. Currently, mLVs are known to transport CSF‐ISF mixtures from the brain to dcLNs, efficiently clearing molecules and immune cells from the cerebral fluid environment (Chachaj et al. 2023). Together with the glymphatic pathway, they form a “lymphatic‐like” network for cerebral metabolic waste clearance.

### 3.2 Functional Roles of the Glymphatic System

The GS is a key structure for maintaining cerebral metabolic homeostasis, efficiently clearing metabolic waste via synergistic actions. With PVS formed by astrocyte endfeet as its core channel, the GS mediates the clearance of neuronal activity‐derived waste (e.g., Aβ) from the brain to the external environment through unidirectional CSF‐ISF flow, thereby inhibiting the onset and progression of CNS diseases (Yamada and Iwatsubo 2024; Lopes et al. 2022). Meanwhile, mLVs further drain GS‐processed waste to the peripheral lymphatic circulation via direct connections to cranial base lymph nodes, completing the clearance process. The synergy between the GS and mLVs forms an essential cerebral waste clearance network, playing an irreplaceable role in maintaining brain microenvironment stability. Similar to the peripheral lymphatic system, mLVs also exert immune surveillance functions. The CNS is a site of highly active and regulated immune surveillance, and the peripheral lymphatic system can mediate cerebral immune monitoring via mLVs (Salvador et al. 2024). Under physiological conditions, abundant immune cells (e.g., dendritic cells, T cells) accumulate around dural sinuses. Ablation of dcLNs or ligation of mLVs reduces dural T cell content, confirming mLVs as the primary conduit for immune surveillance between the CNS and peripheral lymphatic system (Louveau et al. 2015; Aspelund et al. 2015).

#### 3.2.1 The Initiation and Osmosis of Cerebrospinal Fluid

CSF is primarily produced by the choroid plexus in the lateral, third, and fourth ventricles, with its generation dependent on Na/K‐ATPase and aquaporin 1 (AQP1) expressed in choroid plexus epithelial cells (de Laurentis et al. 2021). In glymphatic circulation, CSF permeates deep into the brain parenchyma along the PVS—especially arterial PVS—driven by arterial pulsations derived from cardiac contractions and low‐frequency vascular smooth muscle tension changes (vascular motion) (Beschorner and Nedergaard 2024; Mestre et al. 2020). As a core structural component of the GS, PVS are formed by pia mater extensions accompanying the interstitial spaces of cerebral arteries/veins at all levels and their surrounding vasculature within the brain parenchyma. Located between the endothelial cell basal lamina and the glial limitans (predominantly composed of astrocyte endfeet that cover 60%–98% of cerebral blood vessels (Mathiisen et al. 2010)), PVS enable fluid transport via outer membrane connective tissue and connect to the capillary basement membrane potential spaces (Gouveia‐Freitas and Bastos‐Leite 2021). Astrocyte endfeet envelop blood vessels to form a “perivascular sheath,” with the interprocess channels providing a structural pathway for CSF to enter the brain parenchyma.

#### 3.2.2 Exchange of Interstitial Fluid and Waste Collection

CSF flowing into the PVS is primarily regulated by AQP4 expressed at the end feet of astrocytes (Lynch et al. 2022). It enters the interstitial space through the pia mater and glial cells (Tarasoff‐Conway et al. 2015; Bohr et al. 2022). AQP4 is the main water channel protein in the brain, and its polarization promotes CSF influx into the brain parenchyma (Ishida et al. 2022), regulating brain homeostasis (Peng et al. 2023; Hablitz and Nedergaard 2021; Mogensen et al. 2021). When AQP4 expression shifts from the end feet of astrocytes to the entire astrocytic cell body, it is referred to as AQP4 depolarization (Ding et al. 2023), which can impair the function of GS (Mestre et al. 2018). After entering the brain parenchyma, CSF mixes with the ISF surrounding neurons and glial cells, carrying away metabolic waste products (such as Aβ and tau proteins) through diffusion and convection (Ishida et al. 2022; Nedergaard and Goldman 2016).

#### 3.2.3 Pathways for Waste Excretion

**FIGURE 1:**
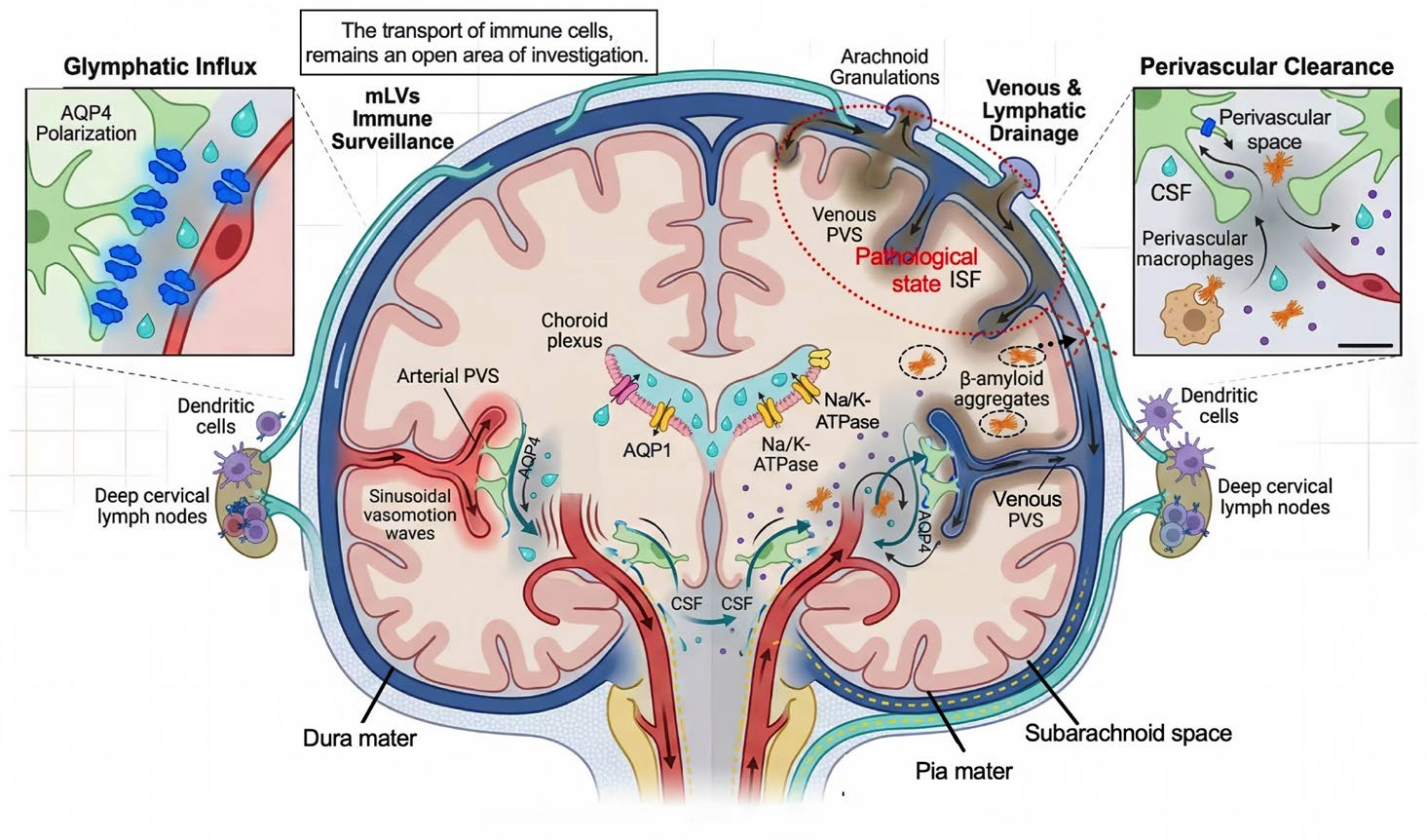
The schematic diagram of the fluid dynamics within the GS (The GS is a key structure for maintaining cerebral metabolic homeostasis, achieving efficient metabolic waste clearance via synergistic actions. In glymphatic circulation, CSF permeates deep into the brain parenchyma along leptomeningeal PVS‐particularly arterial PVS‐driven by arterial pulsations. Upon entering the brain parenchyma, CSF mixes with neuronal and glial ISF, removing metabolic waste via diffusion and convection. The waste‐laden mixed fluid enters venous PVS through AQP4 channels in astrocyte endfeet, gradually converging back to the subarachnoid space along venous blood flow. Ultimately, waste is eliminated from the brain via two pathways: Partial fluid is reabsorbed into venous sinuses through arachnoid granulations, entering systemic circulation; the remainder is drained via skull base lymphatic pathways (e.g., perineural and perivascular spaces) to dcLNs, completing final clearance.).

After the exchange, the mixed solution carrying metabolic waste enters the PVS around the veins through the AQP4 channels at the end feet of astrocytes, gradually converging back to the subarachnoid space along the direction of venous blood flow (Ishida et al. 2022). Ultimately, the waste is expelled from the brain through the following pathways: part of the fluid is reabsorbed into the venous sinuses via the arachnoid granulations, entering the systemic circulation (Benveniste et al. 2019; Pollay 2010). Another portion is drained to the dcLNs through lymphatic pathways at the skull base (such as the spaces along cranial nerves and blood vessels), completing the final clearance (Aspelund et al. 2015). In rodents, waste exits through the perineural pathways of the trigeminal nerve, glossopharyngeal nerve, vagus nerve, and accessory nerve (Lundgaard et al. 2017) (Figure 1).

### 3.3 Factors Influencing the Glymphatic System

#### 3.3.1 Endogenous Mechanical Driving Forces

##### 3.3.1.1 Cerebrospinal Fluid

The continuous generation of CSF in the choroid plexus facilitates its flow from the ventricular system into the subarachnoid space, subsequently moving along the para‐arterial space into the interstitial regions of the brain. The driving force produced by this CSF flow is crucial for the exchange between CSF and ISF within the GS, as well as for the clearance of metabolic products (Jessen et al. 2015).

##### 3.3.1.2 Arterial Pulsation

Studies have shown that unilateral carotid artery ligation reduces arterial pulsation by 50% and concurrently reduces CSF‐brain ISF exchange. Conversely, the adrenergic receptor agonist dopamine increases arterial pulsation by 60% and promotes perivascular CSF‐ISF exchange. These findings suggest that cerebral arterial pulsation is a key driving force of the GS (Benveniste et al. 2019; Plog and Nedergaard 2018; Iliff, Wang, et al. 2013). Kyrtsos and Baras (2015) demonstrated that increased heart rate accelerates amyloid clearance, while vascular sclerosis enhances Aβ deposition, indicating that cardiac pulsation‐derived arterial pulsatility regulates GS clearance function. Vascular sclerosis may impair this process by attenuating arterial pulsation transmission, reducing ISF convective flow, and ultimately compromising GS function. Kress et al. (2014) further confirmed this hypothesis by showing that aging weakens perivascular CSF circulation, accompanied by a 27% reduction in arterial pulsation.

##### 3.3.1.3 Respiratory Movement

From a physiological perspective, rhythmic inspiration generates negative intrathoracic pressure to boost systemic venous return to the right heart. This remodels intracranial venous hemodynamics and diminishes interstitial fluid pressure gradients around cerebral blood vessels, mechanically dilating PVS. Widened PVS act as enlarged channels that allow CSF to infiltrate the brain parenchyma, accelerating GS circulation and the elimination of metabolic waste. Accumulating evidence identifies respiratory movement as a primary driving force of glymphatic clearance.

Clinical observations of patients with obstructive sleep apnea (OSA) further verify this mechanistic connection. Disordered respiratory cycles abolish regular intrathoracic pressure oscillations. Apneic episodes raise intrathoracic pressure, triggering venous distension, PVS constriction, impaired collateral venous drainage, and erratic pressure fluctuations—factors that collectively hinder glymphatic waste clearance. Since respiratory pressure gradients are indispensable for interstitial fluid transport within brain tissue, disturbed respiratory pressure dynamics in OSA patients correspond to defective CSF‐mediated Aβ clearance (Kiviniemi et al. 2016; Ju et al. 2016). A systematic review and meta‐analysis investigating correlations between late‐life dementia and sleep disturbances (insomnia and OSA) confirmed that OSA accelerates the incidence and progression of both AD and vascular dementia, whereas insomnia only exacerbates AD progression (Kitamura et al. 2020). This disparity implies partially overlapping yet distinct pathological pathways underlying the two dementia subtypes. OSA‐related sleep fragmentation and cerebral structural abnormalities compromise glymphatic function and elevate AD susceptibility, a conclusion corroborated by Roy et al. (2022), who reported impaired GS activity among OSA patients.

#### 3.3.2 Physiological State Regulation

##### 3.3.2.1 Sleep

Age‐related declines in sleep quality and disrupted sleep architecture are common precursors to dementia in neurodegenerative diseases. The GS, which clears cerebral protein waste, exhibits peak activity during sleep; its age‐associated degeneration suggests a causal link between sleep disorders and the progression of neurodegenerative dementia (Shokri‐Kojori et al. 2018; Eide et al. 2021; Nedergaard and Goldman 2020). Neural slow waves support memory consolidation, while CSF clears brain metabolic waste. Fultz et al. (2019) used accelerated neuroimaging to characterize human brain physiology and neurodynamics, identifying coherent oscillations in electrophysiology, hemodynamics, and CSF dynamics during nonrapid eye movement (NREM) sleep. Hemodynamic oscillations follow neural slow waves and couple with CSF flow, indicating CSF dynamics are linked to neural and hemodynamic rhythms (Fultz et al. 2019). Lee et al. (2022) assessed GS function in patients with isolated rapid eye movement (REM) sleep behavior disorder (iRBD) via diffusion tensor image analysis along the perivascular space (DTI‐ALPS), with comparisons to healthy controls. Results showed significantly lower DTI‐ALPS indices in iRBD patients, confirming GS dysfunction in this population (Lee et al. 2022). Locus coeruleus‐derived noradrenergic signaling is critical for arousal (Steriade et al. 1993; Carter et al. 2010; Constantinople and Bruno 2011); notably, adrenergic receptor inhibitors modulate arousal and accelerate CSF flow along the GS (Xie et al. 2013). This indicates that sleep–wake states, rather than circadian rhythms, regulate cerebral interstitial volume and thus GS clearance efficiency.

Xie et al. (2013) found that tracer influx along PVS increases significantly during sleep, likely due to a 60% expansion of cerebral interstitial space, which reduces tissue resistance to tracer flow and enhances GS clearance efficiency. Concurrently, elevated ISF convective flux during sleep accelerates Aβ clearance (Xie et al. 2013). Kang et al. (2009) performed sleep–wake studies on freely behaving animals and demonstrated that cerebral ISF Aβ levels correlate positively with wakefulness and negatively with sleep. SD (Sleep deprivation) further accelerates amyloid plaque deposition in APP‐expressing transgenic mice, indicating that sleep–wake behavior regulates Aβ levels and sleep abnormalities may contribute to AD pathogenesis. Holth et al. (2019) investigated the effects of the sleep–wake cycle and SD on ISF and CSF tau protein seeding and spreading. Results showed that awake mice exhibit ~90% higher ISF tau levels than sleeping mice, with a ~100% increase during SD; human CSF tau levels rise by over 50% under SD. Chronic SD also enhances pathological tau spreading in seeding models (Holth et al. 2019). Collectively, the sleep–wake cycle modulates ISF tau levels, and SD elevates tau levels and promotes its pathological spread in ISF and CSF (Holth et al. 2019). Body posture during sleep may affect CSF‐ISF exchange efficiency. Lee et al. (2015) used dynamic contrast‐enhanced MRI and kinetic modeling to quantify CSF‐ISF exchange rates in anesthetized rodents under supine, prone, and lateral postures. Glymphatic transport efficiency was highest in the lateral position, followed by supine; the prone position (mimicking awake upright posture) showed tracer retention and slower clearance (Lee et al. 2015). Lateral and supine postures thus facilitate glymphatic transport and Aβ clearance, with the lateral position optimizing waste removal during sleep (Lee et al. 2015). Tuura et al. (2021) explored correlations between MRI‐measured nocturnal changes in water diffusivity, brain volume, and CSF flow, and high‐density sleep electroencephalogram (EEG) parameters and circadian rhythms. Nocturnal water diffusivity increased, correlating positively with REM sleep proportion and negatively with non‐REM sleep proportion; it also associated with the sleep midpoint (a circadian marker). CSF flow also increased, but this change linked to circadian rhythm markers rather than sleep or diffusivity metrics (Tuura et al. 2021). In a randomized crossover study at the University of Gothenburg (Olsson et al. 2018), healthy adults with normal sleep patterns underwent 5 nights of baseline 8‐h sleep, followed by 5 nights of partial SD (4‐h sleep); 4 participants completed 8 nights of 4‐h sleep. Sleep was monitored via observation, actigraphy, and polysomnography, with CSF samples collected by lumbar puncture postintervention. Analysis of CSF biomarkers (Aβ_38_, Aβ_40_, Aβ_42_, total tau, phosphorylated tau, etc.) revealed no significant correlations between partial SD and amyloid deposition, AD‐related neurodegenerative changes, or astrocyte activation biomarkers (Olsson et al. 2018).

In summary, sleep‐related factors—including the sleep–wake cycle, sleep posture, SD, and sleep disorders (e.g., OSA, RBD)—modulate GS function, thereby regulating cerebral levels, pathological propagation, and abnormal accumulation of Aβ and tau proteins. Notably, conclusions from human SD studies remain inconsistent. Specifically, sleep disorders impair the GS's core function of clearing pathological proteins during sleep, which drives the pathogenesis and progression of AD. Future research should elucidate the molecular mechanisms underlying sleep‐mediated GS regulation; developing sleep‐targeted interventions may provide theoretical support and practical approaches for preventing and slowing the progression of neurodegenerative diseases such as AD.

#### 3.3.3 Exogenous Pharmacological Regulation

##### 3.3.3.1 Anesthetics

Ketamine and xylazine are among the earliest anesthetics used in GS research (Iliff et al. 2012). As a dissociative anesthetic, ketamine induces altered consciousness and analgesia by inhibiting thalamic‐neocortical pain transmission and stimulating brainstem‐limbic circuits; however, it lacks sufficient potency for surgical anesthesia and is typically combined with xylazine, an α2‐adrenergic receptor agonist. Hablitz et al. (2019) evaluated CSF tracer influx into the GS of wild‐type mice under six anesthetic regimens, alongside EEG power, heart rate, blood pressure, and respiratory rate. Results showed that ketamine/xylazine yielded the highest CSF tracer influx, followed by isoflurane (Iso) plus dexmedetomidine and pentobarbital. In contrast, α‐chloralose, Avertin, or Iso alone resulted in lower tracer influx. This study first identified a positive correlation between glymphatic influx and cortical δ‐wave power, and a negative correlation with β‐wave power and heart rate (Hablitz et al. 2019). Gakuba et al. (2018) used MRI and near‐infrared fluorescence imaging to assess the effects of general anesthesia (Iso, ketamine, or ketamine/xylazine) on cerebral CSF circulation in mice. Findings indicated that CSF circulation is primarily active during wakefulness and impaired under general anesthesia, with this effect being most pronounced at high Iso doses (3%) (Gakuba et al. 2018). von Holstein‐Rathlou et al. (2018) injected fluorescent tracers into the cisterna magna of awake mice and those anesthetized with ketamine/xylazine, then analyzed tracer distribution in coronal brain sections. Their data demonstrated higher glymphatic activity under ketamine/xylazine anesthesia (von Holstein‐Rathlou et al. 2018). Groothuis et al. (2007) found that the half‐life of CSF glucose clearance under pentobarbital anesthesia (24 h) was significantly longer than that in unanesthetized mice or those receiving ketamine/xylazine (2–3 h). This suggests that solute clearance from the normal cerebral extracellular space involves multiple physiological pathways and is regulated by anesthetic agents.

Plog et al. (2018) found that increasing plasma osmolality without disrupting the BBB tripled CSF tracer influx into the GS. In APP/PS1 mice, elevated plasma osmolality inhibited wakefulness‐induced glymphatic suppression and enhanced Aβ antibody delivery, increasing antibody binding to Aβ plaques fivefold (Plog et al. 2018). AQP4—a key protein in cerebral waste clearance that regulates synaptic plasticity and neurocognition—was investigated by Yilmaz et al. (2023), who showed that repeated Iso exposure caused more severe brain injury than single exposure. Postanesthesia, cortical AQP4 expression was higher than in the hippocampus and cerebellum, with elevated AQP4 levels negatively correlating with apoptosis, reactive oxygen species, and inflammation (Yilmaz et al. 2023). Prolonged Iso exposure may induce perioperative neurocognitive disorder (PND) via unclear mechanisms; Dong et al. (2024) confirmed that PND mice exhibit impaired GS function, with sluggish glymphatic transport linked to hippocampal inflammatory protein accumulation. Enhancing AQP4 polarization improved glymphatic transport, suppressed neuroinflammation, and reversed cognitive deficits in PND mice (Dong et al. 2024). Benveniste et al. (2017) demonstrated that dexmedetomidine (a norepinephrine‐lowering agent) alone or combined with low‐dose Iso increased rat glymphatic transport by 32%; notably, hippocampal glymphatic clearance efficiency under the combined regimen was six times higher than with Iso alone (Benveniste et al. 2017).

In summary, anesthetics, plasma osmolality, and AQP4 exert complex regulatory effects on the GS. Specifically, ketamine/xylazine anesthesia enhances GS activity and CSF tracer influx, whereas high‐dose Iso and pentobarbital inhibit GS‐mediated CSF circulation and solute clearance; glymphatic influx correlates positively with EEG delta power and negatively with beta power and heart rate. Elevated plasma osmolality (without BBB disruption) boosts GS function and Aβ antibody delivery. Additionally, Iso anesthesia affects AQP4 expression and polarization, with Iso‐induced GS impairment closely associated with PND pathogenesis; enhancing AQP4 polarization can ameliorate GS function and cognitive deficits. Compared with Iso monotherapy, dexmedetomidine plus low‐dose Iso better preserves or enhances GS function, particularly hippocampal clearance efficiency. These findings clarify the regulatory mechanisms of GS function, providing critical references for optimizing GS‐related experimental design and developing PND intervention strategies.

## 4 The Glymphatic System and Alzheimer's Disease

CSF enters the brain parenchyma via arterial PVS and is cleared through venous PVS, identifying PVS as key sites for CSF‐brain parenchyma material exchange. Recent diffusion tensor imaging studies of PVS in AD patients revealed a positive correlation between PVS water molecule diffusion rate and MMSE scores, implying a link between AD pathogenesis and the GS (Taoka et al. 2017). Studies on GS driving forces show that reduced CSF pressure impairs the clearance of substances like Aβ; Schirinzi et al. (2017) confirmed that AD patients have lower CSF pressure than healthy controls, which correlates with Aβ levels, further verifying the GS‐Aβ clearance association. AQP4 depolarization is known to impair GS function, and age‐related AQP4 polarity changes are accompanied by Aβ deposition, suggesting that compromised GS function promotes Aβ accumulation (Zeppenfeld et al. 2017). Additionally, AQP4 gene single nucleotide polymorphisms (SNPs) are associated with cognitive dysfunction in AD patients: rs9951307 and rs3875089 correlate with chronic progressive cognitive decline, while rs3763040 and rs3763043 are linked to rapid progressive decline (Burfeind et al. 2017). However, the specific molecular mechanisms by which AQP4 SNPs regulate GS‐mediated Aβ clearance remain unclear. Collectively, these findings confirm that the GS plays a critical role in AD onset and progression.

### 4.1 The Glymphatic System as a Pathway for Clearing Key Pathological Proteins in AD

**FIGURE 2:**
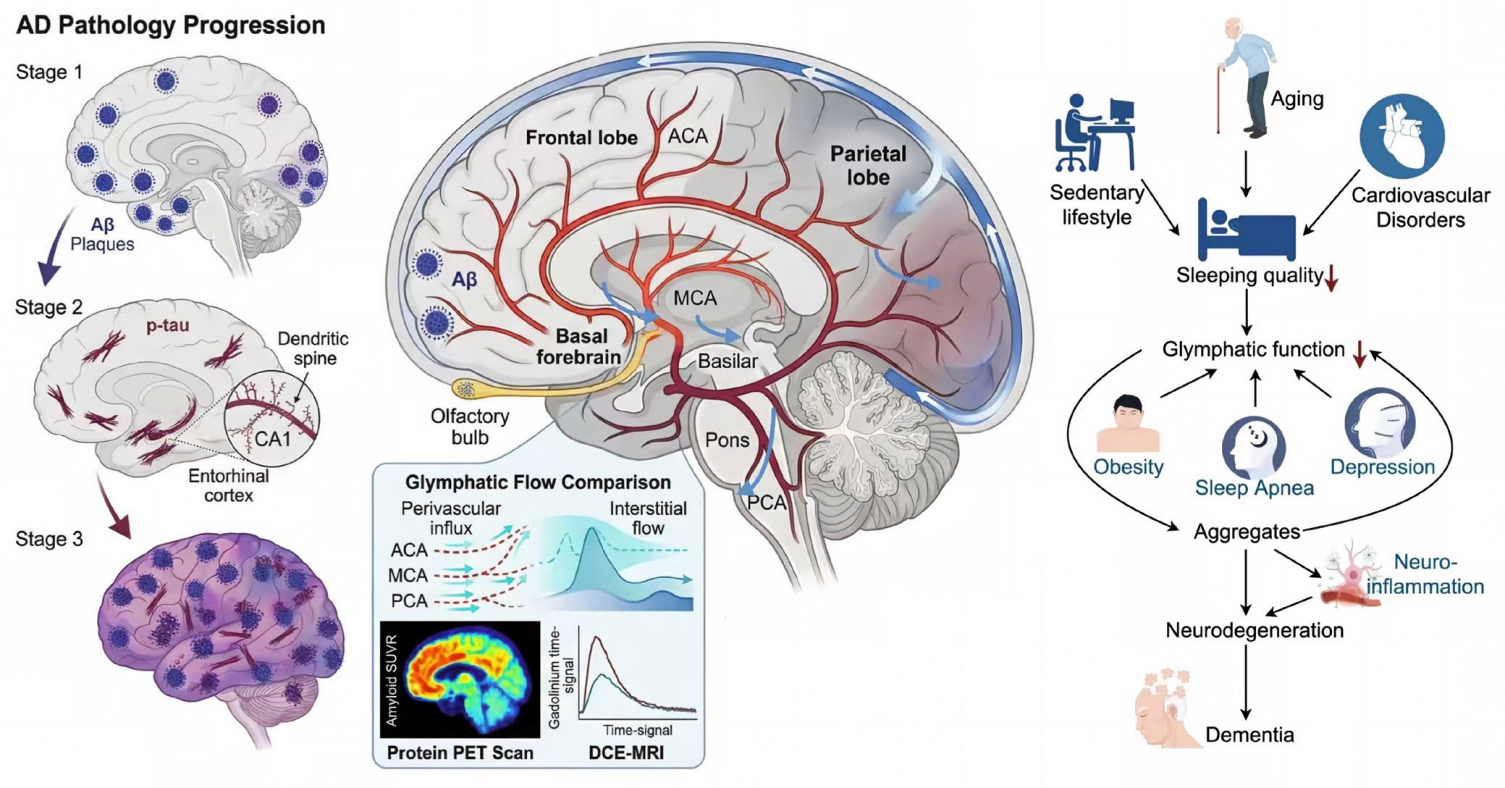
Diagram illustrating the significance of AD pathological protein propagation, the GS, and sleep in dementia prevention (Nedergaard and Goldman 2020). (Overall, pathological protein aggregation in AD initiates in the ventral basal forebrain and midbrain, gradually spreading to the cortex. A key question is the relationship between this pathological spread pattern and the GS‐CSF flow pathways. Neuroimaging studies demonstrate that intrathecally injected contrast agents initially distribute along the cerebral arterial system; while it remains unclear whether these pathogenic proteins accumulate in GS pathways, their distribution closely matches the cerebral lymphatic inflow pathways identified by MRI. Additionally, sleep synergizes with the GS to maintain brain health, highlighting the importance of improving sleep and preserving lymphatic function for dementia prevention.).

The brain employs multiple pathways to clear metabolic wastes such as Aβ, including intracerebral degradation via the ubiquitin‐proteasome system and autophagy‐lysosomal mechanisms (Liu et al. 2021; Do and Baek 2021), transport across the BBB and blood‐CSF barrier, ISF bulk flow, CSF absorption into the circulatory system (Uchida et al. 2023), and clearance through the GS (Rasmussen et al. 2022). CSF enters the brain interstitial space, mixes with cellular ISF to facilitate substance exchange, and transports metabolic wastes from brain tissue to perivenous outflow pathways via a process termed bulk flow. Bulk flow is independent of solute molecular weight and transport distance, enabling efficient and consistent clearance of diverse brain metabolites. The ISF in perivenous pathways is ultimately drained to cervical lymph nodes and incorporated into extracranial lymphatic circulation. In the elderly or AD patients, the choroid plexus undergoes structural alterations (calcification, fibrosis, Aβ deposition), which impair CSF production, reduce CSF turnover, hinder Aβ clearance, and elevate CSF Aβ load. Moreover, amyloid deposition and fibrosis in arachnoid villi further obstruct CSF outflow, establishing a vicious cycle. Accumulating evidence indicates that CSF can drain not only into the bloodstream via arachnoid villi but also into peripheral lymph nodes through cranial nerves and mLVs (Figure 2).

In the APP/PS1 AD model, accumulation of toxic Aβ species (e.g., soluble oligomers) reduces glymphatic transport regardless of Aβ plaque deposition. Notably, GS dysfunction precedes amyloid deposition (Peng et al. 2016), implying that GS decline may serve as an early indicator of AD. Huang et al. (2024) assessed brain GS activity via diffusion tensor imaging‐along the perivascular space (DTI‐ALPS). Results showed reduced ALPS indices in patients with AD dementia, prodromal AD, and preclinical AD; this reduction correlated with positron emission tomography‐measured Aβ burden and Aβ‐positive conversion. Importantly, the ALPS index decreased before CSF Aβ_42_ reached the positive threshold, confirming that impaired GS function is an early pathological feature of AD. Patel et al. (2019) injected Cypate‐labeled tau protein into GS‐deficient K14‐VEGFR3‐Ig transgenic mice and their wild‐type littermates. At 48 and 72 h postinjection, transgenic mice exhibited higher cerebral tau retention and delayed peripheral tau clearance, demonstrating the GS's critical role in tau clearance. Additionally, tau pathology models showed impaired CSF‐ISF exchange and AQP4 polarization; treatment with the AQP4 inhibitor TGN‐020 further inhibited CSF‐ISF exchange and reduced tau clearance efficiency, suggesting the GS is an important pathway for cerebral tau clearance in AD (Harrison et al. 2020). Zeppenfeld et al. (2017) analyzed AQP4, Aβ_1‐42_, and GFAP distribution/expression in the frontal cortex of cognitively healthy individuals and histopathologically confirmed AD patients using immunofluorescence and Western blotting. AQP4 expression correlated with aging across all subjects, whereas perivascular AQP4 localization correlated specifically with AD status independent of age (Zeppenfeld et al. 2017). After adjusting for age, loss of perivascular AQP4 localization was associated with increased Aβ burden and advanced Braak stages (Zeppenfeld et al. 2017).

#### 4.1.1 Meningeal Lymphatic Vessels and AD

Da Mesquita et al. (2018) found that ablating or damaging mLVs significantly reduces GS clearance efficiency, indicating GS function is regulated by mLVs; they are functionally linked via CSF despite no obvious anatomical connection. MRI in humans shows intrathecal tracers appear sequentially in brain parenchyma, mLVs, and dcLNs, revealing a continuous CSF drainage pathway and confirming mLVs as downstream components of the GS (Zhou et al. 2020). Known cerebral lymphatic drainage pathways include nasal (cervical) lymphatics, basal mLVs, and dorsal mLVs (Louveau et al. 2015; Ahn et al. 2019; Hsu et al. 2019). mLVs run parallel to dural arteries/veins (abundant near dural venous sinuses, with lymph flow opposite to superior sagittal sinus venous flow (Albayram et al. 2022; Kuo et al. 2018)), may extend through skull foramina with cranial nerves (supporting a perineural drainage pathway (Albayram et al. 2022; Ma et al. 2017)), and are mainly confined to the dura mater (though a few studies report their presence in the pia mater and subarachnoid lymphatic‐like membranes (Mollgard et al. 2023)). Composed of lymphatic endothelial cells, mLVs express specific markers (e.g., LYVE‐1, PROX1, Podoplanin, VEGFR3 (Louveau et al. 2015)). The traditional “immune privilege” of the CNS, based on lack of lymphatic surveillance and isolation from peripheral immunity (Negi and Das 2018), is challenged by mLVs. As a bridge between central and peripheral immunity, mLVs drain CNS antigens and immune cells to dcLNs, critical for CNS immune surveillance (Ahn et al. 2019; Rasmussen et al. 2018). T cells, B cells, and dendritic cells migrate to dcLNs via mLVs with CSF (Louveau et al. 2018). Kaminski et al. (2012) confirmed monocytes can migrate from the CNS to dcLNs through the cribriform plate/nasal lymphatics. dcLNs are T cell activation sites, where dendritic cells process antigens to promote effector T/B cell activation and proliferation (Louveau et al. 2018; Engelhardt et al. 2016; Jian et al. 2019; Karman et al. 2004).

Louveau et al. (2018) demonstrated that mLVs possess two core characteristics: first, they facilitate the drainage of CSF components; second, they enable immune cells to enter lymph nodes in a C‐C chemokine receptor type 7 (CCR7)‐dependent manner. Unlike lymphatic vessels in other tissues, meningeal lymphatic endothelial cells do not dilate during inflammation and exhibit unique transcriptional features (Louveau et al. 2018). This functional specificity allows mLVs to act as a bridge between central and peripheral immunity, playing a crucial role in CNS immune surveillance (Ahn et al. 2019). For instance, in animal models of multiple sclerosis, ablation of mLVs reduces pathological damage and inhibits the inflammatory response of brain‐reactive T cells, providing new insights for the treatment of this disease (Louveau et al. 2018). In the context of AD, accumulating evidence supports the involvement of mLVs in disease progression. Da Mesquita, Papadopoulos, et al. (2021) found that impaired mLV drainage exacerbates microglial inflammatory responses in AD mice. Nauen and Troncoso (2022) used immunohistochemistry to examine resected cervical cancer tissues and inguinal lymph nodes, revealing Aβ‐labeled cells in lymph nodes—with a significantly higher abundance in cervical lymph nodes than in inguinal lymph nodes—supporting the hypothesis that the lymphatic system aids Aβ clearance in the human brain. Pappolla et al. (2018). further confirmed this using Tg2576 transgenic mice (overexpressing mutant β‐amyloid precursor protein, APP). They found that melatonin treatment increased soluble monomeric Aβ_40_ (and trended upward for Aβ_42_) in cervical and axillary lymph nodes, while reducing Aβ_40_/Aβ_42_ oligomers in the brain. These results indicated that the GS, in coordination with mLVs, participates in brain Aβ clearance, and abnormalities in this clearance function may contribute to cerebral amyloidosis (Pappolla et al. 2018). CCR7, a key molecule mediating mLV‐dependent immune cell migration, is closely linked to mLV and GS function. Da Mesquita, Herz, et al. (2021) observed reduced CCR7 expression in meningeal T cells of aged mice; CCR7 deficiency led to decreased glymphatic influx and cognitive impairment. In 5xFAD transgenic mice, CCR7 deficiency further exacerbated neurovascular damage, reduced glymphatic clearance efficiency, overactivated microglia, and increased brain Aβ deposition (Da Mesquita, Herz, et al. 2021).

However, conflicting findings have emerged regarding the role of mLVs in AD. Studies on human autopsy samples found no Aβ deposition in mLVs associated with the superior sagittal sinus or their vicinity (Goodman et al. 2018). Antila et al. (2024) showed that regulating mLVs (either inducing atrophy or proliferation via blocking or overexpressing vascular endothelial growth factor‐C (VEGF‐C)) had no effect on brain Aβ deposition or behavioral scores in AD mice. Instead, meningeal amyloid deposits were mostly confined to bridging veins connecting the brain and large dural venous sinuses (Antila et al. 2024). This contradicts previous studies, and researchers speculate that gradual changes in mLVs induced by VEGF‐C may trigger compensatory CSF outflow dynamics, while differences in Aβ deposition patterns between AD mice and human patients may also contribute to this discrepancy (Antila et al. 2024). Therefore, the role of mLVs in the pathophysiological mechanisms of AD requires further exploration.

#### 4.1.2 Perivascular Clearance Pathways and AD

The perivascular clearance pathway, a glymphatic drainage route in the CNS, is closely linked to cerebral amyloid angiopathy (CAA) associated with Aβ. Vascular aging impairs this pathway's efficiency, causing insoluble Aβ to deposit in cerebral vessel walls and form CAA. A study of 330 cerebral small vessel disease (CSVD) patients showed that reduced DTI‐ALPS values correlated significantly with white matter hyperintensities (WMH); patients with poorer baseline glymphatic function had faster WMH progression over 1.5 years of follow‐up (Zhang, Zhou, et al. 2021). Moreover, some CSVD biomarkers are associated with AD‐related Aβ/tau pathology, and WMH increases AD risk (Liu et al. 2018). CAA, a type of CSVD characterized by vascular Aβ deposition, is classified into type 1 (Aβ deposits in capillaries, inducing perivascular inflammation) and type 2 (Aβ deposits in leptomeningeal and cortical arteries). Clinically, CAA is a major cause of intracerebral hemorrhage and strongly correlates with cognitive impairment and dementia, though its pathophysiological mechanisms remain unclear (Greenberg et al. 2020).

Chen et al. (2022) found that in type 1 CAA rats, CSF flows faster along arterial PVS but fails to fully exchange with brain ISF despite unimpaired influx into the brain, reducing overall GS transport capacity. Neck imaging revealed that some CSF bypassed ISF exchange, reaching dcLNs via carotid arteries with delayed arrival time, indicating disrupted crosstalk between the brain GS and meningeal lymphatic system in type 1 CAA rats. Thus, CAA impairs GS function, exacerbates Aβ accumulation, and indirectly promotes AD onset and progression (Chen et al. 2022). Recent studies indicate distinct basement membrane routes for fluid inflow and outflow in the perivascular clearance pathway (Albargothy et al. 2018). For inflow, CSF enters the brain parenchyma along the basement membrane between the pia mater and glial limitans, penetrates arterial basement membranes, descends into capillary basement membranes, and mixes with ISF via AQP4. For outflow, ISF and solutes (e.g., Aβ) ascend along capillary epithelial‐glial limitans basement membranes, then flow out via arterial smooth muscle cell basement membranes; Aβ is cleared by smooth muscle cells and perivascular macrophages during this process. The basement membrane is a key component of this pathway, regulated by APOE and influenced by prenatal high‐fat diets, atherosclerosis, Aβ deposition, and AD immune complexes (Sun et al. 2018).

### 4.2 Glymphatic System, Glial Cells and Neuroinflammation in AD

With deepening research, the GS has emerged as a vital bridge linking CNS homeostasis and neuroimmunology. Glial cells serve as the structural and functional core of GS; accordingly, this section systematically elaborates the multifaceted roles of distinct glial populations, as well as the reciprocal crosstalk between glymphatic transport impairment and neuroinflammation, which jointly drive AD pathological progression.

#### 4.2.1 The Vascular PVS and Perivascular Macrophages in Central Neuroinflammation

As the fundamental anatomical channel of the glymphatic system, PVS undergo prominent structural remodeling under inflammatory conditions, including immune cell infiltration, pro‐inflammatory mediator accumulation, and enlarged cavity volume. Existing evidence confirms that PVS constitute the primary route for lymphocyte drainage and peripheral monocyte recruitment into the brain parenchyma (Zou et al. 2024). Perivascular macrophages, a specialized glia‐derived immune subset residing within PVS, exert prominent anti‐inflammatory effects against stress‐induced neuroinflammation and oxidative/nitrosative damage. They dominate the uptake and clearance of inflammatory macromolecules within interstitial fluid, thereby maintaining GS transport efficiency. In the absence of functional perivascular macrophages, inflammatory debris accumulates within PVS and blocks CSF‐ISF exchange, initiating the early stage of glymphatic dysfunction.

#### 4.2.2 Bidirectional Interaction Between Glymphatic Dysfunction and Microglial Overactivation

Impaired glymphatic clearance and microglial hyperactivation form a self‐amplifying vicious cycle. Deficient CSF circulation reduces the brain's capacity to eliminate pro‐inflammatory cytokines and chemokines while lowering anti‐inflammatory IL‐10 levels in the CNS, which spontaneously triggers microglial activation. Accumulated inflammatory agonists further activate the NLRP3 inflammasome in microglia, amplifying the secretion of inflammatory mediators (Heneka et al. 2025; Hansen et al. 2018). Conversely, sustained microglia‐derived inflammation disrupts glymphatic transport. Neutrophils recruited during chronic inflammation release neutrophil extracellular traps, aggravating interstitial tissue inflammation and further obstructing perivascular drainage pathways (Hidalgo et al. 2022). Single‐cell sequencing data further verified that meningeal lymphatic drainage insufficiency indirectly drives microglial enlargement and excessive activation, accompanied by lysosome hyperproliferation and abnormal S100A8/A9 release of damage‐associated molecular patterns (Kim et al. 2025). Depletion or pharmacological inhibition of microglia partially rescues glymphatic transport defects, confirming microglia as a central mediator linking GS clearance and neuronal inflammation. Excess IL‐6 secreted by overloaded microglia induces inhibitory synaptic loss and cerebral functional decline, revealing a key inflammatory effector in this pathological loop (Das Neves et al. 2024).

#### 4.2.3 Astrocyte AQP4 Polarization: Core Glial Structure Sustaining Glymphatic Circulation

Astrocytes and their polarized AQP4 channels are the indispensable core glial component of the glymphatic system. Aβ plaque deposition, a hallmark pathological feature of AD, directly triggers astrocyte phenotypic transformation and AQP4 depolarization, which collapses the structural basis for CSF‐ISF exchange (Yang et al. 2011). Loss of perivascular AQP4 polarity severely suppresses interstitial waste clearance, accelerating Aβ aggregation and inflammatory substance retention. This astrocyte dysfunction amplifies neuroinflammation in return, creating a closed pathological loop between glial structural damage, glymphatic failure, and amyloid pathology.

#### 4.2.4 Meningeal Lymphatic Impairment Mediates Glial Inflammation and White Matter Injury

mLVs primarily undertake the peripheral drainage of CSF and metabolic waste to dcLNs. Different from the glial‐central glymphatic machinery, mLVs function as an upstream auxiliary clearance pathway. Severe mLV dysfunction leads to toxic metabolite buildup in the brain, subsequently triggering cascade activation of microglia and loss of mature oligodendrocytes. Oligodendrocyte depletion further disrupts white matter integrity and exacerbates neuroinflammation, indirectly aggravating glymphatic circulation disorders (Das Neves et al. 2024). Notably, meningeal lymphatic drainage defects act as an initiating factor rather than the core mechanism of glymphatic failure; the ultimate functional collapse of GS mainly depends on impaired astrocytic AQP4 polarization and microglial inflammatory blockade.

#### 4.2.5 Pathogenic Trigger: *Porphyromonas gingivalis* Disrupts Glial Function and Glymphatic Clearance

Periodontopathic *Porphyromonas gingivalis* has been validated as an extrinsic risk factor for cerebral Aβ deposition and AD progression (Kamer et al. 2015; Noble et al. 2009). Clinical observations indicate that AD patients complicated with periodontitis display faster cognitive deterioration, and dental treatment alleviates memory deficits in over half of mild AD patients with gingival lesions (Ide et al. 2016; Rolim et al. 2014). Current mechanistic evidence remains insufficient to fully clarify this correlation; animal‐based findings hypothesize that intracranial translocation of *Porphyromonas gingivalis* disturbs microglial circadian rhythm, disrupts sleep‐dependent glymphatic clearance, and ultimately promotes cerebral Aβ aggregation (Takayama et al. 2016). The accumulated Aβ further induces astrocyte AQP4 depolarization, forming a complete pathogenic cascade from exogenous infection to glial and glymphatic dysfunction.

#### 4.2.6 The Vicious Cycle of Glymphatic Dysfunction‐Neuroinflammation as a Therapeutic Target for AD

**FIGURE 3:**
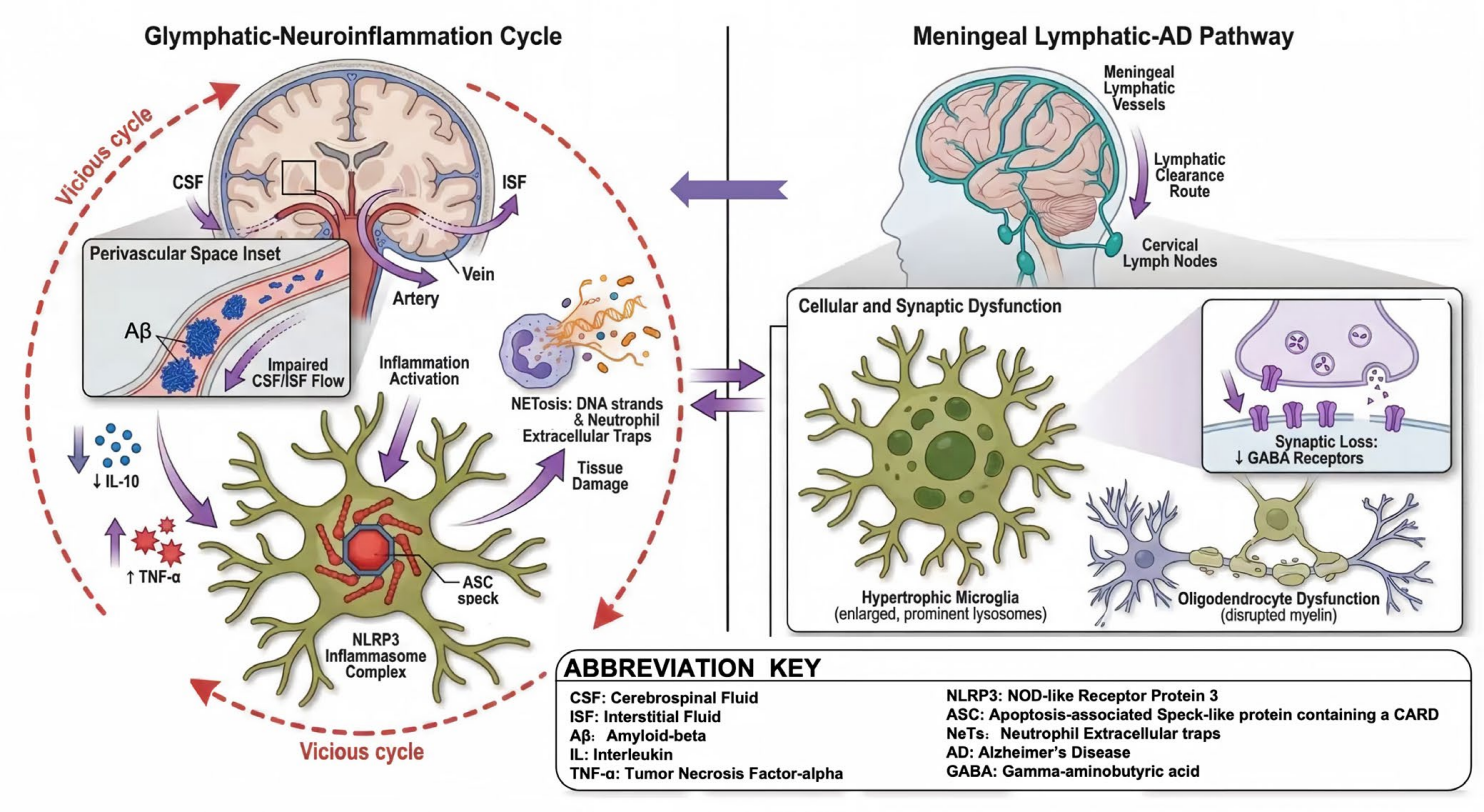
Key Players of The Glymphatic System in Neuroinflammation with AD (Impaired glymphatic drainage leads to the accumulation of metabolic wastes such as β‐amyloid, which activates the NLRP3 inflammasome, elevating pro‐inflammatory TNF‐α and reducing anti‐inflammatory IL‐10. This triggers microglial activation and neutrophil NETosis, exacerbating brain tissue damage, which in turn further suppresses glymphatic drainage, forming a self‐reinforcing vicious cycle.).

The reciprocal interaction between glial‐mediated glymphatic impairment and progressive neuroinflammation represents a core pathological mechanism underlying AD (Figure 3). Breaking this vicious loop provides a novel therapeutic direction for AD intervention, including three major strategies: restoring astrocytic AQP4 polarity via pharmacological agents, promoting mLV regeneration to improve peripheral waste drainage, and selectively suppressing key pro‐inflammatory signaling pathways in microglia. Nevertheless, multiple molecular details of the crosstalk between inflammatory cytokines and glial glymphatic structures remain unclarified. Future research is required to decode the precise regulatory network between cytokines and AQP4 polarization, supporting the development of precise glia‐targeted glymphatic therapies for AD.

### 4.3 The Role of the Glymphatic System and the Blood–Brain Barrier in AD

The BBB, a critical physiological barrier separating blood from the CNS, consists of brain microvascular endothelial cells, intercellular tight junctions, basement membrane, pericytes, and astrocytic endfeet (Tang et al. 2021). It has two core functions: blocking blood‐borne harmful substances from entering brain tissue and selectively transporting peripheral nutrients into the CNS, thereby precisely maintaining cerebral microenvironmental homeostasis (Serlin et al. 2015). Thus, BBB structural integrity is essential for preserving brain environmental stability. The BBB and GS synergize in waste clearance via partially overlapping mechanisms (Kress et al. 2014). When metabolic wastes are distant from the BBB or exceed its transport capacity, the GS compensates and can enhance BBB transport efficiency (Verheggen et al. 2018). Dynamic Aβ clearance across the sleep–wake cycle is tightly linked to the crosstalk between these two pathways. Moreover, BBB disruption induces basement membrane thickening and PVS reduction, impairing glymphatic function (Goulay et al. 2017). Disrupted BBB‐GS balance interferes with protein clearance and deposition, accelerating the onset and progression of AD and cerebral small vessel disease (CSVD), and fostering a vicious cycle between the two pathways (Tian et al. 2022).

## 5 Targeted Therapeutic Strategies for Alzheimer's Disease Based on CNS Glymphatic Drainage Pathways

### 5.1 Pharmacological Therapy

Drugs must cross the BBB to reach the CNS from the systemic circulation. Direct CSF injection has limited brain permeability, often resulting in insufficient therapeutic efficacy (Lohela et al. 2022). To address this, strategies targeting BBB disruption (e.g., virus‐derived peptides and toxins) have been proposed to enhance brain delivery efficiency (Chan et al. 2021; Zhang, Niu, et al. 2025; Xu et al. 2025). However, BBB integrity impairment raises safety concerns, particularly with repeated treatments (Li, Chen, et al. 2025). Additionally, most intravenous drugs undergo hepatic first‐pass metabolism, further reducing bioavailability. Thus, to overcome CNS drug delivery challenges, numerous researchers are focusing on GS‐mediated drug delivery (Wang et al. 2025) (Table 2).

**TABLE 2:** Pharmacological and nonpharmacological effects on glymphatic system function.

Intervention	Model	Function/glymphatic system function	References
Low‐dose infusions of mononuclear human umbilical cord blood cells	APPswe/PSEN1dE9 (APP/PS1) transgenic mice	Cognitive impairment↓ Amyloid‐β (Aβ)plaques↓ Amyloidogenic amyloid precursor protein (APP) processing↓ Reactive microgliosis↓	Darlington et al. (2013)
Human umbilical cord blood cells	Male, retired breeder rats were subjected to the multiple microinfarction model	Improves glymphatic function by reversing multiple microinfarction induced delay in the penetration of cerebrospinal fluid (CSF) into the brain parenchyma MiR‐126 expression in serum↑ Aquaporin‐4 (AQP4) expression↑	Venkat et al. (2020)
n‐3 polyunsaturated fatty acids (PUFAs)	Fat‐1 transgenicmice andoral administration of fish oil	Promote interstitial Aβ clearance while beneficial effects were abolished in AQP4‐knockout mice n‐3 PUFAs protect the AQP4 polarization in the affected brain region after Aβ injection	Ren et al. (2017)
L‐3‐n‐butylphthalide (L‐NBP)	APP/PS1 transgenic mice	L‐NBP promoted the perivascular drainage of Aβ via increased cerebral pulsation, which could be inhibited by propranolol Improved the perivascular AQP4 localization.	Zhang, Li, et al. (2021)
Plasma hyperosmolality	Male global AQP4‐knockout (*Aqp4* ^*−/−*^) mice on a C57BL/6 background. Male 6‐month‐old APP/PS1^+/−^ mice.	Inhibitied glymphatic transport that characterizes the awake state Reversed glymphatic suppression	Plog et al. (2018)
Multisensory 40 Hz stimulation	Viptm1^(cre)Zjh^/J (Jax 010908) were crossed to 5XFAD to generate Vasoactive Intestinal Peptide Causes recombination (VIP)‐Cre 5XFAD heterozygous mice. 5xFAD (Tg 6799) crossed with C57BL/6 mice to generate off spring	VIP interneurons upregulated transcripts related to neuropeptide synthesis and secretion Neuropeptides are released in a frequency‐dependent manner VIP interneurons facilitate glymphatic clearance by regulating arterial pulsatility. Inhibitied glymphatic clearance abolished the removal of amyloid.	Murdock et al. (2024)
Low‐intensity 40 hertz blue light	5xFAD mice with a B6/SJL genetic background	Restoration of glial water channel AQP‐4 polarity and a reduction in hippocampal lipid accumulation. Mediated through the activation of the ventral Lateral Geniculate Nucleus/Intergeniculate Leaflet‐Retinorecipient (vLGN/IGL‐Re) visual circuit.	Wu et al. (2025)
Repetitive Transcranial magnetic stimulation (rTMS)	5xFAD mice	The amount of tracer trapped in the medial prefrontal cortex of 5xFAD mice was significantly elevated, whereas rTMS treatment ameliorated the accumulation of tracer Significant reduction of tracer was detected in the dura mater and the deep cervical lymph nodes (dcLNs) of the 5xFAD mice as compared to their wild‐type littermates, whereas rTMS treatment significantly prevented such changes.	Lin et al. (2021)
rTMS	Cerebral amyloid angiopathy (CAA) model (Tg‐SWDI) mice	The tracer intensity in the corpus callosum and cerebral cortex of mice in the CAA group was significantly higher than rTMS group. The polarization level of AQP4 in the cerebral cortex of mice in the rTMS group was significantly greater than that in the CAA group.	Li, Yang, et al. (2025)
Continuous theta‐burst stimulation (CTBS)	APP/PS1 transgenic mice	CTBS could increase glymphatic fluid transport, especially CSF and interstitial fluid exchange, mediated by improved AQP4 polarization.	Wu et al. (2022)
GABA or its GABAA receptor antagonist through cisterna magna injection	C57BL/6J mice AQP4 knockout mouse model	GABA promotes glymphatic clearance in an AQP4‐dependent manner by activating the GABAA receptor.	Wu et al. (2023)
40 Hz transcranial vibration stimulation (TVS)	Human brain	40 Hz TVS promoted the synchronization of overall brain activity with CSF, an effect that 30 Hz or 50 Hz TVS did not replicate.	Kong et al. (2025)
Focused ultrasound treatment in combination with microbubbles (FUS‐MB)	5xFAD mice	FUS‐MB enhanced solute Aβ clearance from brain, but not plaques, to CSF space and dcLNs. dcLNs ligation exaggerated memory impairment and progress of plaque formation and also the beneficial effects of FUS‐MB upon Aβ removal through CSF‐lymphatic routes.	Lee et al. (2020)
Deep cervical lymphaticovenous anastomosis (dcLVA)	Patients with Alzheimer's Disease (AD)	Following assessment using the Mini‐Mental State Examination (MMSE) and Montreal Cognitive Assessment (MoCA), it was observed that the behavioral performance, cognitive function, and memory of patients with AD demonstrated significant improvement.	Lee et al. (2020)

#### 5.1.1 Exogenous Carriers and Cell Preparations With Indirect GS Regulatory Effects

PLGA nanoparticles and human umbilical cord blood cells (HUCBCs) exert neuroprotective effects via incidental improvement of glymphatic drainage, rather than direct targeting of GS molecular components. Subcutaneous cervical injection of PLGA nanoparticles loaded with indocyanine green achieves efficient intracerebral transport through cerebral lymphatic vessels, suppressing glioblastoma proliferation in mice; this particle transport route relies on intact perivascular glymphatic pathways (Zhao et al. 2020). As for HUCBC intervention, long‐term cell infusion alleviates cognitive decline, Aβ plaque deposition, and microglial overactivation in AD model mice, and reverses memory impairment in multiple microinfarction rats (Darlington et al. 2013; Venkat et al. 2020). Concurrent alterations including elevated perivascular AQP4, increased serum miR‐126, and decreased cerebral TGF‐β imply a hypothetical mechanism: HUCBCs may remodel cerebral white matter and rescue compromised glymphatic clearance function. We emphasize that this causal link remains unconfirmed and requires further verification.

#### 5.1.2 Small‐Molecule Nutrients Directly Modulating AQP4‐Dependent Glymphatic Clearance

n‐3 PUFAs serve as a typical agent that directly acts on core GS machinery. Experiments in AQP4‐knockout mice confirm that n‐3 PUFAs accelerate interstitial Aβ clearance strictly through AQP4‐mediated glymphatic transport (Ren et al. 2017). Omega‐3‐rich fish oil upregulates BBB efflux transporter LRP1, reduces cerebral soluble and insoluble Aβ deposits, and promotes peripheral Aβ drainage (Yan et al. 2020; Wen et al. 2024), which validates its direct regulatory role on GS clearance pathways.

#### 5.1.3 Botanical Extracts Indirectly Enhancing Glymphatic Circulation

L‐3‐n‐butylphthalide (L‐NBP) is a neuroprotective botanical extract with indirect GS‐improving capacity. In 3xTg‐AD mice, L‐NBP shifts APP metabolism toward the nonamyloidogenic pathway to reduce Aβ generation (Peng et al. 2010). Follow‐up mechanistic research further demonstrates that L‐NBP boosts cerebral vascular pulsatility, facilitates CSF tracer inflow and perivascular waste drainage in APP/PS1 mice. We hypothesize that enhanced vascular pulsation restores sluggish glymphatic clearance, which partially accounts for its anti‐AD effects, rather than direct modulation of AQP4 or other GS proteins (Zhang, Li, et al. 2021).

#### 5.1.4 Endogenous Hormones Facilitating GS‐Mediated Aβ Efflux

Melatonin likely promotes glymphatic Aβ clearance via peripheral lymphatic drainage (this remains a speculative hypothesis). In Tg2576 mice, melatonin elevates soluble monomeric Aβ levels in cervical and axillary lymph nodes while lowering cerebral Aβ oligomers, implying it may accelerate GS‐dependent Aβ export from the brain to peripheral lymphatic tissue (Galvani et al. 2024). Still, direct molecular evidence connecting melatonin to AQP4 polarization or perivascular transport is currently lacking.

Distinct from classic AD therapies that solely target pathological proteins (Aβ or tau), pharmacological interventions restoring glymphatic circulation represent an innovative therapeutic direction. The core advantage of GS‐targeted strategies lies in reconstructing the brain's inherent metabolic waste clearance capacity: this approach not only attenuates neurotoxic protein buildup at the source but also relieves AD‐related comorbidities including cerebral microcirculation disorder and sleep dysfunction, matching the multi‐target, holistic intervention concept for neurodegenerative diseases. Notably, multiple unresolved bottlenecks restrict clinical translation of GS‐regulating drugs: the complete molecular cascades of glymphatic dysfunction in AD are not fully clarified, most candidate agents lack specific GS‐targeting selectivity, and human clinical trials remain insufficient. We put forward the following perspective for future research: in‐depth mechanistic exploration of GS dysfunction, development of brain‐specific delivery systems, and precise patient stratification will jointly advance the translation of glymphatic‐targeted AD therapeutics, which may provide new strategies to slow disease progression and rescue cognitive impairment in AD patients.

### 5.2 Noninvasive Intervention Pathways

Noninvasive neuromodulation and physical interventions modulate glymphatic circulation by regulating cerebrospinal fluid‐interstitial fluid (CSF‐ISF) exchange, AQP4 polarization and meningeal lymphatic outflow, representing a safe auxiliary strategy to restore impaired glymphatic clearance without systemic drug exposure. Plog et al. (2018) verified that elevated plasma osmolarity (without BBB destruction) expanded PVS drainage and tripled CSF tracer influx. In AD mice, osmotic regulation suppressed sleep–wake cycle‐related glymphatic inhibition, rescued glymphatic dysfunction, and enhanced Aβ antibody binding to plaques approximately fivefold.

Gamma sensory stimulation is another GS‐regulating modality. Multisensory 40 Hz gamma stimulation accelerates CSF inflow and ISF efflux in 5XFAD mice; blockade of glymphatic clearance completely eliminates its amyloid‐clearing capacity (Murdock et al. 2024). Consistently, low‐intensity 40 Hz blue light normalized AQP4 polarity, boosted cerebral waste drainage, and reduced hippocampal lipid deposition, alleviating cognitive impairment in young and aged AD mice. Combined administration with anti‐Aβ antibodies further facilitated soluble Aβ removal, which hypothesizes its potential value as adjuvant AD therapy (Wu et al. 2025).

Repetitive transcranial magnetic stimulation (rTMS) and its subtype continuous theta‐burst stimulation (CTBS) improve glymphatic transport via neuromodulation. rTMS restores parenchymal glymphatic and mLV drainage, mitigates Aβ deposition and cognitive decline in 5XFAD and CAA mice by repolarizing perivascular AQP4 (Lin et al. 2021; Li, Yang, et al. 2025). Two‐photon imaging further confirmed that CTBS facilitates CSF‐ISF exchange through GABA‐AQP4 signaling cascades to enhance glymphatic clearance and reduce cerebral amyloid burden (Wu et al. 2022, 2023). Similarly, 40 Hz transcranial vibration stimulation (TVS) couples whole‐brain neural activity with CSF circulation to facilitate glymphatic clearance, whereas 30 and 50 Hz stimulation fail to exert such effects (Kong et al. 2025).

Focused ultrasound combined with microbubbles (FUS‐MB) accelerates soluble Aβ translocation from brain parenchyma to CSF and dcLNs. Ligation of dcLNs aggravated amyloid pathology and memory loss, confirming that its amyloid‐clearing effect mainly relies on glymphatic‐CSF peripheral drainage pathways (Lee et al. 2020). Transcutaneous auricular vagus nerve stimulation (taVNS) improves CSF circulation and alleviates vascular cognitive dysfunction in rodent models, which provides a novel noninvasive intervention candidate for AD (Choi et al. 2022).

In summary, all above noninvasive approaches exert favorable glymphatic regulatory effects in preclinical animal models. However, several translational limitations remain to be addressed: optimized stimulation parameters for human subjects, long‐term safety profiles, and standardized operation protocols are still lacking. We propose that future work should prioritize clinical trial validation, human‐specific device development, and combinatorial regimens with Aβ/tau‐targeted medications to achieve synergistic clearance‐improving effects for AD patients with glymphatic dysfunction.

### 5.3 Invasive Intervention Pathways

Invasive interventions targeting meningeal and cervical lymphatic drainage mainly include surgical anastomosis and animal‐based lymphangiogenic gene therapy, which can robustly remodel glymphatic outflow pathways but carry inherent operational risks. dcLVA originated from accidental clinical observation: a patient with facial lymphedema obtained relieved AD‐related cognitive symptoms after lymphatic surgery, which generated the hypothesis that dcLVA may improve AD pathology by restoring peripheral cerebral waste drainage (Onoda et al. 2023). A single 84‐year‐old AD case showed obvious cognitive improvement postoperatively, which drew widespread attention to this domestic exploratory surgical technique (Onoda et al. 2023). Driven by preliminary clinical observations, dcLVA has been carried out in numerous domestic hospitals. Nevertheless, we emphasize that current clinical evidence is extremely limited: only isolated case reports are available, without large‐sample, controlled high‐quality clinical trials. Multiple unresolved critical questions restrict its standardized clinical application: the optimal AD patient subgroups suitable for surgery, whether the intervention can merely relieve symptoms or reverse underlying amyloid pathology, and its efficacy for patients complicated with hydrocephalus or drug‐refractory moderate–severe AD remain unclarified (Xie et al. 2024).

AD and idiopathic normal pressure hydrocephalus (iNPH) share overlapping pathological features including disrupted glymphatic circulation and Aβ accumulation; abnormal CSF dynamics jointly drive progressive neuronal injury, and misdiagnosis between the two diseases frequently occurs (Reeves et al. 2020; Zhao et al. 2018; Olazaran et al. 2013). Rigorous multicenter clinical trials and unified diagnostic and therapeutic criteria are urgently required to verify the clinical value of dcLVA and gain international recognition. Other invasive strategies are only validated in preclinical models. Cranial bone manipulation enhances mLV formation and meningeal lymphatic drainage to reduce amyloid deposition in 5XFAD mice, yet clinical evidence is absent (Lu et al. 2025). Adeno‐associated virus 8‐mediated VEGF‐C injection induces meningeal lymphangiogenesis, suppresses neuroinflammation, and improves glymphatic drainage in hepatic encephalopathy models, which offers an experimental tool to remodel lymphatic outflow (Hsu et al. 2021). Chronic epidural electrode implantation stimulates dural lymphatic sprouting and promotes CSF influx into glymphatic spaces in mice, but long‐term implantation trauma limits its clinical transformation (Hauglund et al. 2020).

Overall, invasive glymphatic‐targeted therapies face prominent translational barriers. Surgical procedures carry risks of hemorrhage, infection, and peripheral nerve injury, demanding refined surgical techniques and biocompatible materials. Furthermore, therapeutic efficacy observed in rodent models may be attenuated in humans due to interspecies BBB differences and individual glymphatic functional heterogeneity. In addition, the complete molecular regulatory network of glymphatic dysfunction in AD has not been fully elucidated, hindering the development of precise invasive interventions. Collectively, substantial mechanistic and clinical research is still needed to advance this field.

## 6 Conclusion and Outlook

Since the GS was first identified more than a decade ago, abundant anatomical, molecular, and imaging evidence has verified its presence in the human brain, yet the translational gap between preclinical findings and clinical practice remains substantial. Many candidate therapeutic strategies targeting glymphatic clearance are still debated and lack sufficient clinical evidence to support widespread application.

Extensive research in anatomy, molecular medicine, and imaging has confirmed the existence of the GS in the human brain, which facilitates fluid drainage to cervical lymph nodes via CSF‐ISF exchange within the cranial cavity. While the anatomical structure of the GS remains incompletely characterized, and most studies have focused on rodent models with a paucity of large‐scale clinical trials, investigations into the structural and functional features of GS pathways have shed new light on the highly organized fluid transport and clearance mechanisms in the CNS. This provides novel directions and hope for the diagnosis and treatment of AD.

However, several critical questions remain unresolved: clarifying the causal relationship between GS dysfunction and AD etiology/pathological changes, and identifying key factors that promote the maturation and repair of mLVs. Currently, noninvasive interventions have shown promising results in preclinical studies; future efforts should prioritize translating their safety and efficacy to human trials, alongside developing noninvasive GS imaging and assessment techniques to enable large‐scale clinical research. Notably, the 2022 clinical application of dcLVA for AD in China achieved promising outcomes, potentially opening a new therapeutic avenue. This represents a notable contribution of microsurgery to neurology, yet numerous issues require further validation. Foremost, optimized surgical indications remain ill‐defined (Pan 2025): it is urgent to determine which AD subgroups benefit—all AD patients, only moderate‐to‐severe cases, or those with preoperatively confirmed deep cervical lymphatic drainage obstruction? Additionally, AD and iNPH share overlapping pathological and clinical features (e.g., Aβ deposition, GS dysfunction, progressive dementia) and CSF dynamic abnormalities (Reeves et al. 2020). Studies indicate iNPH may increase AD risk, with ~26% of iNPH patients exhibiting AD‐related pathology (Zhao et al. 2018), and misdiagnosis of iNPH as AD is not uncommon (Olazaran et al. 2013). Thus, it remains unproven whether dcLVA improves AD by alleviating iNPH‐like symptoms.

Theoretically, if dcLVA enhances GS function, it may promote the clearance of soluble Aβ and tau; however, it is unlikely to affect insoluble (and toxic) proteins, nor reverse neuronal loss or synaptic reduction—raising questions about its long‐term efficacy. Consequently, clarifying dcLVA's therapeutic mechanisms for AD, its short‐ and long‐term effects, pre‐ and postoperative assessment methods, surgical indications/contraindications, standardized procedures, and follow‐up protocols is critical for its clinical application in AD management.

Overall, the field of glymphatic research is still in its early translational stage. Rigorous large‐cohort clinical trials, standardized imaging evaluation systems, and high‐quality controlled studies for invasive surgical interventions are urgently required to resolve current controversies and validate the clinical value of glymphatic‐targeted therapies for AD.

## Author Contributions

L.F., L.Y., and X.L.: conceptualization, methodology, resources, supervision, project administration. B.S., W.W., and L.F.: writing – review and editing. M.L., Y.L., S.L., X.J., and D.Y.: investigation, visualization. Y.Q., L.Y., X.L., and L.F.: funding acquisition. All authors approved the submitted version.

## Funding

This study was supported by the National Natural Science Foundation of China (82274538), the Natural Science Foundation of Shandong (ZR2023QH159 and ZR2020MH357), the Taian City Science and Technology Innovation Development Project (2023NS469), the Shandong Province Traditional Chinese Medicine Science and Technology Project (Z20243705), Clinical Research Special Project of Shanghai Yangpu District Health System (Grant No. YPM202532), and the Shandong Province Medical Health Science and Technology Development Plan Project (202313011384).

## Ethics Statement

The authors have nothing to report.

## Conflicts of Interest

The authors declare no conflicts of interest.

## Data Availability

The data that support the findings of this study are available on request from the corresponding author. The data are not publicly available due to privacy or ethical restrictions.
